# Phosphine-Assisted Forced Hot-Air Treatment for Phytosanitary Disinfestation of *Bactrocera correcta* in Mango Fruit

**DOI:** 10.3390/insects17060614

**Published:** 2026-06-10

**Authors:** Changyao Shan, Hang Zou, Li Li, Wenze Cao, Baishu Li, Jiajiao Wu, Qiang Xu, Haijun Liu, Tao Liu

**Affiliations:** 1 Institute of Equipment Technology, Chinese Academy of Quality and Inspection & Testing, No. A3, Gaobeidianbeilu, Chaoyang District, Beijing 100123, China; 34424808@student.murdoch.edu.au (C.S.); 25a0904188@cjlu.edu.cn (H.Z.); lili@caiq.org.cn (L.L.); libaishu@163.com (B.L.); 2 Harry Butler Institute, Murdoch University, Perth, WA 6150, Australia; 3 College of Life Sciences, China Jiliang University, Hangzhou 310018, China; 4 Guangzhou Customs District Technology Center, No. 66 Huacheng Avenue, Tianhe District, Guangzhou 510623, China; lh_hui@163.com (W.C.); wujj@iqtcnet.cn (J.W.); xuqiangbj@foxmail.com (Q.X.)

**Keywords:** phosphine, forced hot-air treatment, synergistic effect, phytosanitary disinfestation, postharvest quality, *Bactrocera correcta*, mango

## Abstract

*Bactrocera correcta* is an important quarantine pest of mango, and the development of phytosanitary treatments that achieve quarantine security without compromising fruit quality remains a major challenge in fresh-fruit trade. Heat treatment is a residue-free phytosanitary option, but the temperatures required to control fruit flies often approach the tolerance limits of tropical fruit, leaving a narrow margin between quarantine security and commodity injury. In this study, a phosphine (PH_3_)-assisted forced hot-air treatment was evaluated for the phytosanitary disinfestation of *B. correcta* in mango fruit. The developmental progression of *B. correcta* in mango fruit was characterized, the heat tolerance of different developmental stages was compared, and the efficacy of PH_3_ followed by forced hot-air treatment (PH_3_→Heat) against eggs was quantified using probit time–mortality analysis. Large-scale confirmatory validation and postharvest quality assessment were then conducted. Eggs were identified as the most heat-tolerant stage. PH_3_ pre-fumigation significantly enhanced forced hot-air treatment, with 0.7 g m^−3^ PH_3_ providing the most practical improvement at quarantine-relevant endpoints. According to this schedule, LT_99.9968_ was reduced by 44 min for heat treatment alone, from 269.0 to 224.5 min, and the large-scale validation yielded no survivors. Postharvest quality evaluation showed that PH_3_→Heat did not adversely affect firmness, total soluble solids, or titratable acidity during shelf life. These results demonstrate that PH_3_-assisted forced hot-air treatment is a technically feasible and commercially promising phytosanitary strategy for mango fruit.

## Simple Summary

Effective phytosanitary treatments are required to control quarantine pests in mango without compromising fruit quality. *Bactrocera correcta* is a key pest whose eggs develop within the fruit and are difficult to eliminate. In this study, we evaluated phosphine followed by hot-air treatment as a combined approach. Eggs were identified as the most heat-tolerant stage and selected as the target for treatment development. The addition of phosphine prior to heat treatment reduced the time required for effective control from 269.0 to 224.5 min. In large-scale validation tests, both heat treatment alone and the combined treatment resulted in zero survivors, although the combined treatment achieved this outcome in a shorter time. Fruit quality measurements showed no adverse effects on firmness, total soluble solids, or titratable acidity during storage, although a transient increase in respiration rate was observed shortly after treatment. These findings indicate that phosphine-assisted hot-air treatment is a feasible and practical option for improving phytosanitary treatment efficiency in mango while maintaining postharvest quality.

## 1. Introduction

The rapid expansion of international fresh-fruit trade has greatly increased the importance of reliable phytosanitary treatments that can achieve quarantine security without compromising commodity quality. *Bactrocera correcta* (Bezzi, 1916) is a destructive tephritid pest of tropical and subtropical fruit and has been listed as a quarantine pest by many importing countries [1].

In China, *B. correcta* was intercepted in 3271 batches of imported fruit from 28 countries between 2003 and 2013 [1]. Its wide host range, including mango, and its repeated interception in traded fruit make it a continuing biosecurity concern. The reported distribution and spread of *B. correcta* in southwestern China substantially overlap with the main mango-growing regions in China, which are mainly located in tropical and subtropical areas such as Yunnan, Guangxi, Hainan, Sichuan, Guangdong, Guizhou, Fujian, and Taiwan [2,3]. Current phytosanitary options, including fumigation, heat treatment, cold treatment, and irradiation, each have important limitations [4,5,6]. Methyl bromide remains restricted because of its ozone-depleting properties and phytotoxicity risk, cold treatment is often too slow for commercial logistics and can damage chilling-sensitive fruit, and irradiation still faces regulatory and market barriers. Heat treatment is widely accepted and chemically residue-free, but the temperatures required to kill fruit flies often approach the tolerance limits of tropical fruit, creating a narrow margin between insect mortality and fruit injury [7,8,9].

Against this background, combination phytosanitary treatments have attracted increasing attention because they may reduce treatment intensity while maintaining efficacy. Phosphine (PH_3_) is an attractive candidate for such systems because it has low residue, relatively low phytotoxicity, and established use in postharvest pest control, but when used alone it is generally too slow for rapid quarantine application in fresh produce [10]. Previous studies have shown that PH_3_ can interact synergistically with other fumigants and that combining heat with an additional physiological stressor, as in controlled-atmosphere heat systems, can reduce the thermal burden required for quarantine security [11,12,13]. The biological basis for using PH_3_ as a pre-treatment is supported by studies on stored-product insects, particularly *Tribolium castaneum* (Herbst, 1797) and *Rhyzopertha dominica* (Fabricius, 1792). Recent work has linked PH_3_ response in these species with mitochondrial dihydrolipoamide dehydrogenase, lipid-mediated energy reserves, respiratory metabolism, oxidative stress, and mitochondrial protection [14,15,16,17]. These findings suggest that PH_3_ exposure can reduce insect vitality and environmental stress resistance by disrupting energy metabolism and cellular stress responses. Such physiological debilitation may make insects less able to maintain cellular protection and recovery during a subsequent thermal challenge. More recently, phosphine fumigation followed by forced hot-air treatment was shown to improve control of *Bactrocera dorsalis* (Hendel, 1912) in dragon fruit [18,19]. However, it remains unknown whether a similar strategy can be developed for *B. correcta* in mango fruit, which developmental stage defines the true treatment target under this system, and whether improved disinfestation can be achieved without impairing postharvest quality.

The present study addresses these questions by developing and evaluating a PH_3_-assisted forced hot-air treatment system for *B. correcta* in mango fruit. Relative to previous work, this study makes three main contributions. First, it identifies the most heat-tolerant developmental stage of *B. correcta* within mango fruit and establishes eggs as the critical target for treatment development [7,20,21]. Second, it demonstrates, through probit-based time–mortality analysis, that PH_3_ pre-fumigation can significantly enhance forced hot-air treatment and that 0.7 g m^−3^ PH_3_ provides the most practical improvement at quarantine-relevant mortality endpoints [18]. Third, it shows that this combined treatment can achieve zero survivors under large-scale validation while maintaining the principal postharvest quality attributes of mango fruit during shelf life [8,9]. These findings extend phosphine-assisted combination treatment from previously studied host–pest systems to a new commodity and quarantine pest combination, and provide a technically feasible basis for reducing the thermal burden of mango phytosanitation.

Accordingly, we investigated the development of *B. correcta* in mango fruit, identified the most heat-tolerant stage under forced hot-air treatment, evaluated the efficacy of PH_3_ followed by forced hot-air treatment using probit time–mortality analysis, conducted large-scale confirmatory validation, and assessed the effects of the selected treatment on mango postharvest quality. These results extend phosphine-assisted heat treatment to a new host–pest system and provide a practical framework for developing shorter and more commodity-compatible phytosanitary treatments for mango fruit.

## 2. Materials and Methods

### 2.1. Insects, Fruit and Fumigant

A *B. correcta* colony was established from larvae initially collected from infested mangoes in Yuanjiang City, Yunnan Province, China (101°39′ to 102°22′ E, 23°18′ to 23°55′ N), in 2024, and was maintained in a quarantine laboratory in acrylic rearing cages (120 × 60 × 70 cm). Adults were fed with fresh fruit slices and a solid mixture of sucrose and hydrolyzed yeast (3:1). Approximately 15 days later, eggs were collected by placing perforated plastic cups in the adult cages, and larvae were reared on an artificial diet. All life stages were maintained at 25 ± 2 °C and 65 ± 5% relative humidity (RH), with a 12:12 h dark:light photoperiod.

Mango fruit (*Mangifera indica* L. cv. Jinhwang) were harvested at commercial maturity from an orchard in Guangxi, China. The fruit were transported to the laboratory within 24 h of harvest. Healthy fruit free from visible defects, disease symptoms, and mechanical injury, and uniform in size and color were selected for the experiments. The weight of each fruit ranged from approximately 450 to 550 g.

Ammonia-free PH_3_ gas with 86% purity was generated by reacting aluminum phosphide tablets (Nippon Kasei Co., Ltd., Chuo-Ku, Tokyo, Japan) with 5% (*v*/*v*) sulfuric acid solution. The generated PH_3_ gas was transferred into a 1 L gas sampling bag and maintained at the same temperature as the fumigation treatment before use. The impurity of the source gas was taken into account when calculating the dosing volume required to achieve the target concentration.

### 2.2. Development Test

**Figure 1:**
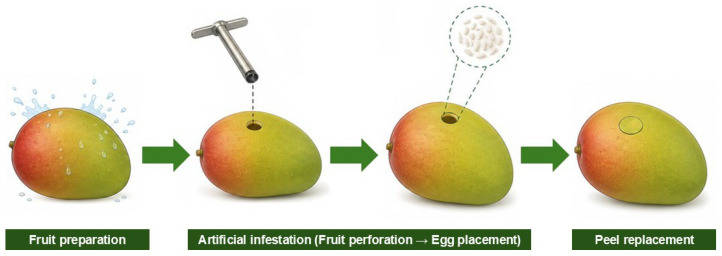
Schematic illustration of the artificial infestation procedure of mango fruit with *Bactrocera correcta*.

To determine the developmental progression of *B. correcta* in mango fruit, healthy mangoes were washed with clean water and held at room temperature for 24 h to allow temperature equilibration before infestation. Each fruit was then artificially infested using a stainless steel coring punch. A circular opening, approximately 3 mm deep, was made in the mango peel, and approximately 50 freshly collected eggs were placed into the opening. The peel plug was then carefully replaced to restore the original fruit surface. A schematic illustration of the artificial infestation procedure is shown in Figure 1.

After infestation, the mangoes were transferred to an environment-controlled chamber and maintained under the same rearing conditions as described above. From day 1 to day 9 after infestation, fruit were dissected daily to determine the developmental stage of *B. correcta* within the fruit. Eggs, 1st-, 2nd-, 3rd-instar larvae, and pupae recovered from dissected fruit were counted, and the proportion of each developmental stage was calculated based on the total number of recovered individuals.

Three replicates were conducted for the development test. Based on the developmental profile obtained, mango fruit incubated for 1, 3, 4, and 5 days after infestation were selected to represent eggs, 1st-, 2nd-, and 3rd-instar larvae, respectively, for the subsequent heat-tolerance and PH_3_→Heat treatment assays.

### 2.3. Heat Tolerance of B. correcta Developmental Stages Under Forced Hot-Air Treatment

**Figure 2:**
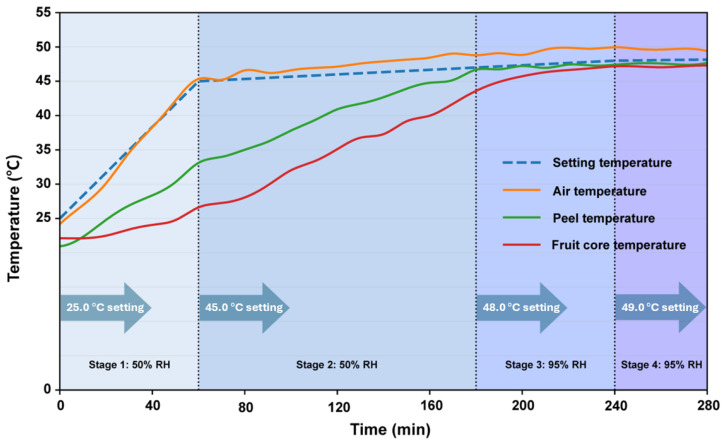
Temperature and relative humidity (RH) profile of air, peel, and fruit core during forced hot-air treatment of mango fruit under program-controlled heating conditions.

To compare the relative heat tolerance of different developmental stages of *Bactrocera correcta* under phytosanitarily relevant temperatures, mango fruit artificially infested with eggs were maintained under rearing conditions for 1, 3, 4, and 5 days to obtain eggs, 1st-, 2nd-, and 3rd-instar larvae, respectively. Infested fruit were then subjected to forced hot-air treatment in a programmable heat-treatment chamber (Chongqing Well Testing Instrument Co., Ltd., Chongqing, China) under a program-controlled stepped heating profile. The forced hot-air treatment followed a four-stage programmable heating profile. In stage 1 (0 to 60 min), the chamber temperature was increased from 25 to 45 °C at 50% RH. In stage 2 (60 to 180 min), the set temperature was further increased from 45 to 48 °C while RH was maintained at 50%. In stage 3 (180 to 240 min), the set temperature was increased from 48 to 49 °C and RH was raised to 95%. In stage 4 (after 240 min), the chamber was maintained at 49 °C and 95% RH until the fruit core reached the target treatment temperature. During treatment, temperature recorders (Ellab A/S, Hillerød, Denmark) were inserted into the fruit to monitor changes in peel and core temperatures. The fruit equipped with the core-temperature recorder was used as the indicator fruit, and the core temperature was continuously monitored throughout the treatment. Once the core temperature of the indicator fruit reached the designated treatment temperature of 46 or 47 °C, the treatment chamber was opened immediately, and the treated fruit were removed. A representative temperature and relative humidity profile recorded during forced hot-air treatment is shown in Figure 2.

After treatment, fruits were immediately cooled in water at 20 °C for 30 min until the core temperature dropped below 30 °C. Treated fruits were then maintained under rearing conditions for survival assessment. Fruit containing eggs and 1st-instar larvae were held for 7 days and then dissected. Individuals that developed into later larval stages were considered survivors. Fruit containing 2nd- and 3rd-instar larvae were held for 2 days, and larvae that did not respond when prodded with a blunt probe were considered dead. Mortality was calculated for each developmental stage based on the number of insects recovered from treated and control fruit. Three independent replicates were conducted for each treatment.

### 2.4. Efficacy Assay of PH_3_ Followed by Forced Hot-Air Treatment Against B. correcta Eggs

After eggs had been identified as the most heat-tolerant stage, infested mango fruit containing eggs were used to evaluate the efficacy of PH_3_ followed by forced hot-air treatment. PH_3_ fumigation was conducted in 6 L glass fumigation containers maintained at 20 °C. Each container held three infested mango fruit, corresponding to a loading ratio of approximately 25% (weight/volume, *w*/*v*). Designated volumes of phosphine gas were injected to achieve target concentrations of 0.7, 1.4, and 2.1 g m^−3^. During fumigation, PH_3_ concentrations were measured at 30 min after injection and again at the end of fumigation. After 3 h fumigation, the containers were opened and the fruit were ventilated in a fume hood for 2 h.

Following fumigation and aeration, the treated fruit were transferred to the forced hot-air treatment chamber and subjected to the same program-controlled heating profile as described in Section 2.3. For the heat-only treatment, infested fruit were directly subjected to forced hot-air treatment without prior fumigation. For heat treatment alone and each PH_3_→Heat treatment, fruits were treated for 130, 140, 150, 160, 170, 180, 190, 200, 210, 220, 230, or 240 min. After heat treatment, fruits were immediately cooled in water at 20 °C for 30 min until the core temperature dropped below 30 °C.

Treated fruits were then maintained under rearing conditions for 7 days and subsequently dissected for survival assessment. Individuals that developed into later larval stages were considered survivors. Untreated infested fruit maintained under the same environmental conditions were used as controls. Three independent replicates were conducted for each treatment. The mortality data obtained from the different heat-treatment durations were used for subsequent efficacy analysis of heat treatment alone and PH_3_→Heat against *B. correcta* eggs.

### 2.5. Large-Scale Confirmatory Test

Based on the results of the efficacy assay and probit analysis, large-scale confirmatory validation was conducted to assess the selected schedules of heat treatment alone and PH_3_→Heat against *B. correcta* eggs in mango fruit. Naturally infested mango fruit were used in the validation experiment. Healthy mango fruit were exposed to ovipositing adult *B. correcta* under rearing conditions to allow natural infestation.

For each treatment, a total of 140 mango fruit were loaded into the treatment system, of which 120 naturally infested fruit were used for large-scale confirmatory validation. Simultaneously, 20 uninfested fruit were retained for postharvest quality evaluation. In addition, 10 naturally infested fruit were maintained as untreated controls for estimation of infestation level. The selected validation schedules were forced hot-air treatment alone for 269.0 min, and 0.7 g m^−3^ PH_3_ fumigation for 3 h followed by forced hot-air treatment for 225 min. PH_3_ fumigation was conducted at 20 °C in a 280 L stainless steel fumigation chamber (85 × 55 × 60 cm), with a loading ratio of about 25.0% (*w*/*v*). After fumigation, the chamber was opened and the fruit were ventilated in a fume hood for 2 h. Thereafter, the fruit were subjected to the same program-controlled forced hot-air treatment profile as described in Section 2.3. Fruit assigned to the heat-only treatment were transferred directly to the heat-treatment chamber without prior fumigation. After heat treatment, fruits were transferred to a temperature-controlled environment at 20 ± 2 °C for 30 min until the core temperature dropped below 30 °C.

After treatment, all naturally infested fruits were maintained under rearing conditions for 7 days and then dissected for survival assessment. Individuals that developed into later larval stages were considered survivors. Because direct counting of all eggs in treated fruit was impractical at this validation scale, the estimated total number of treated insects was calculated based on the infestation rate in the corresponding untreated control fruit and the number of treated fruit. Treatment efficacy was estimated on the basis of zero survival in treated fruit.

### 2.6. Postharvest Quality Evaluation

The 20 uninfested healthy mango fruit treated in parallel with each large-scale confirmatory validation were used for postharvest quality evaluation. In addition, 20 uninfested fruit from the same batch were retained as untreated controls. Thus, three groups were included for quality assessment: untreated control, forced hot-air treatment alone, and PH_3_→Heat. The treatment schedules used for quality evaluation were the same as those used in the large-scale confirmatory validation, namely forced hot-air treatment alone for 269.0 min, and 0.7 g m^−3^ PH_3_ fumigation for 3 h followed by forced hot-air treatment for 225 min. After treatment, fruit were transferred to a temperature-controlled environment at 20 ± 2 °C for shelf-life evaluation, and quality assessments were conducted at 1 and 7 d after treatment. Three independent replicates were conducted for each treatment at each sampling time.

Fruit quality parameters evaluated included respiration rate, firmness, total soluble solids (TSS), and titratable acidity (TA). For respiration measurements, three fruit were placed in a 6 L glass container, and 0.4 mL headspace gas samples were collected after 2 h incubation at the storage temperature. CO_2_ concentrations were determined using gas chromatography equipped with a thermal conductivity detector (GC-TCD). Gas analysis was performed using an 80/100 Porapak Q column at 70 °C with H_2_ as the carrier gas, and respiration rate was expressed as mg CO_2_ kg^−1^ h^−1^.

Fruit firmness was measured using a texture analyzer (TA-XT2i; Stable Micro System, Godalming, UK) with a force resolution of 0.001 N and a force accuracy of 0.025%. Firmness was measured on the equatorial plane of the fruit using a penetration test. A cylindrical probe with a diameter of 5 mm was used at a penetration depth of 5 mm and a speed of 2 mm s^−1^. The firmness of each sample was recorded from the force–distance curve based on the peak force, and the mean value was calculated.

For TSS and TA determinations, juice was extracted from the fruit flesh. TSS was measured using a handheld digital refractometer (GMK-701R; G-won Hitech Co., Ltd., Seoul, Republic of Korea). For TA determination, the juice was measured using an acidity meter (GMK-855; G-won Hitech Co., Ltd., Seoul, Republic of Korea). TSS and TA values were measured in triplicate and recorded. These parameters were used to compare the effects of heat treatment alone and PH_3_→Heat on mango fruit during the post-treatment shelf-life period.

### 2.7. Statistical Analysis

Proportional mortality data were corrected using Abbott’s formula [22]. The means and standard errors (SEs) of replicates were calculated using Microsoft Excel (Microsoft 365 App for Enterprise). Differences among treatments were analyzed using SPSS 27 statistical software (IBM Corp., Armonk, NY, USA), and mean comparisons were conducted using Duncan’s multiple range test. Differences were considered statistically significant at *p* < 0.05. Probit analysis was performed using POLO PLUS software, version 2.0 (LeOra Software, Berkeley, CA, USA).

The synergistic ratio (SR) was used to describe the enhancement effect of PH_3_ pre-fumigation on forced hot-air treatment and was calculated according to Equation (1):

(1) $$SR=\frac{{LT}_{99.9968}\;of\;forced\;hot\text{-}air\;treatment\;alone}{{LT}_{99.9968}\;of\;{PH}_{3}\to Heat}$$

Accordingly, SR = 1 indicates an additive effect, SR < 1 indicates antagonism, and SR > 1 indicates synergism.

For large-scale confirmatory tests with zero survivors, the estimated efficacy (1 − *P*u) was calculated using Equation (2):

(2) $$1-Pu={\left(1-C\right)}^{1/n}$$

where *C* represents the confidence level, *P*u is the acceptable survival level, and *n* is the number of test insects.

Postharvest quality data, including respiration rate, firmness, TSS, and TA, were analyzed by two-way ANOVA in SPSS, with treatment and storage time as the main factors. Mean comparisons were conducted using Tukey’s HSD test at *p* < 0.05.

## 3. Results

### 3.1. Bactrocera correcta Development in Mango

**Figure 3:**
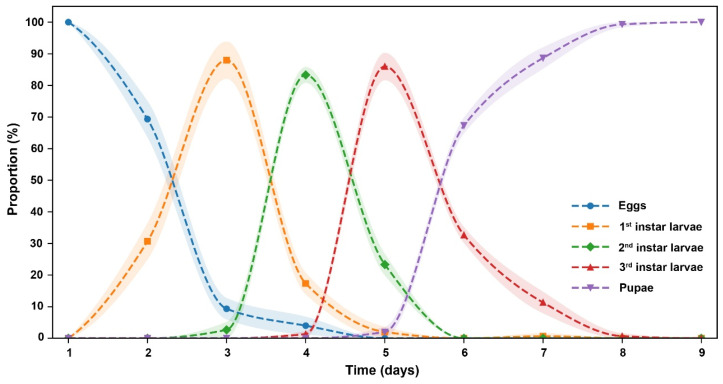
Proportions of *Bactrocera correcta* at different developmental stages in infested mango fruit from day 1 to day 9 after infestation. Data are expressed as means ± SE, shown as error bands.

The proportions of *B. correcta* at different developmental stages recovered from dissected infested mango fruit after 1 to 9 d of incubation are shown in Figure 3. Because the larval instars of *B. correcta* are morphologically similar, the different developmental stages were classified based on the morphological characteristics reported by Shan et al. (2023) [1]. Eggs predominated during the first 2 d of incubation, accounting for 100.00% of individuals on day 1 and 69.33% on day 2. Egg hatch began after 1 d of incubation, and the proportion of first-instar larvae increased to 30.67% on day 2 before reaching its maximum of 88.00% on day 3. The second-instar larvae initially appeared in small numbers on day 3 and became the dominant instar on day 4, accounting for 83.33% of the recovered insects. Third-instar larvae were first detected on day 4 and became predominant on day 5, reaching 86.00%. Pupae appeared from day 5 onwards and increased rapidly thereafter, accounting for 67.33% of individuals on day 6 and more than 88% from day 7 onwards. By day 9, all recovered individuals had reached the pupal stage. On this basis, mango fruit incubated for 1, 3, 4, and 5 d after infestation were selected to represent eggs, first-, second-, and third-instar larvae, respectively, for subsequent heat and PH_3_→Heat treatment assays.

### 3.2. Heat Tolerance of Different Life Stages of B. correcta

Heat treatment temperatures around 46 to 47 °C are commonly used in phytosanitary heat-treatment schedules for tephritid fruit flies in tropical fruit [23,24,25,26]. For mango fruit, vapor heat-treatment schedules have been proposed or adopted in which treatment efficacy is based on fruit pulp or core temperatures reaching approximately 46.5 to 47.5 °C, followed by a defined holding period to achieve quarantine security [27]. Therefore, 46 °C and 47 °C were selected in this study to compare the heat tolerance of different developmental stages of *B. correcta* under temperatures relevant to practical phytosanitary treatment development.

**Figure 4:**
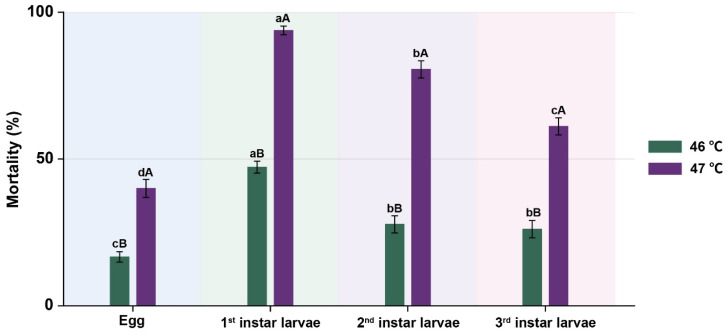
Mortality of different developmental stages of *Bactrocera correcta* after heat treatment at 46 and 47 °C. Different lowercase letters indicate significant differences among developmental stages within the same temperature treatment, whereas different uppercase letters indicate significant differences between temperatures within the same developmental stage (*p* < 0.05).

Mortality of *B. correcta* differed significantly among developmental stages after heat treatment at both temperatures (Figure 4). At 46 °C, mortality was highest in first-instar larvae (47.22%), followed by second-instar larvae (27.78%) and third-instar larvae (26.11%), whereas eggs showed the lowest mortality (16.67%). The mortality of second- and third-instar larvae did not differ significantly from each other, but both were significantly lower than that of first-instar larvae and higher than that of eggs. At 47 °C, mortality increased significantly across all developmental stages. First-instar larvae again showed the highest mortality (93.81%), followed by second-instar larvae (80.56%), third-instar larvae (61.11%), and eggs (40.00%). For each developmental stage, mortality at 47 °C was significantly higher than that at 46 °C, indicating that the heat response of *B. correcta* was highly temperature-sensitive within this range. Eggs consistently showed the lowest mortality at both temperatures, indicating that they were the most heat-tolerant stage under the tested conditions. Therefore, eggs were selected as the target stage for subsequent heat and PH_3_→Heat treatment assays.

### 3.3. Toxicity Assay

Probit analysis showed that PH_3_ pre-fumigation reduced the exposure time required for forced hot-air treatment of *B. correcta* eggs in mango fruit (Table 1). The probit models fitted the mortality data well, with heterogeneity values below 1.0 for all treatments, indicating a good fit of the time–mortality response data. For heat treatment alone, the estimated LT_90_, LT_99_, LT_99.99_, and LT_99.9968_ values were 162.762, 197.473, 255.516, and 269.030 min, respectively. In comparison, all PH_3_→Heat treatments significantly reduced the estimated LT_90_ and LT_99_ values compared with heat treatment alone. The LT_90_ values decreased to 136.923, 130.784, and 127.820 min after pre-fumigation with 0.7, 1.4, and 2.1 g m^−3^ PH_3_, respectively, while the LT_99_ values decreased to 165.616, 164.264, and 161.330 min, respectively.

At the higher mortality endpoints, only the 0.7 g m^−3^ PH_3_→Heat treatment showed a statistically significant reduction compared with heat treatment alone. This treatment reduced LT_99.99_ and LT_99.9968_ from 255.516 and 269.030 min to 213.422 and 224.526 min, respectively. The 1.4 and 2.1 g m^−3^ PH_3_→Heat treatments also reduced the point estimates of LT_99.99_ and LT_99.9968_, but these reductions were not clearly separated from heat treatment alone at the 95% confidence level. The SRs based on LT_99.9968_ were 1.198, 1.137, and 1.149 for 0.7, 1.4, and 2.1 g m^−3^ PH_3_→Heat, respectively. These results indicate that the enhancement effect of PH_3_ pre-fumigation was not simply concentration-dependent, and that 0.7 g m^−3^ PH_3_ was sufficient to achieve the most consistent and statistically supported reduction in the required heat exposure at the quarantine-relevant endpoints.

**Figure 5:**
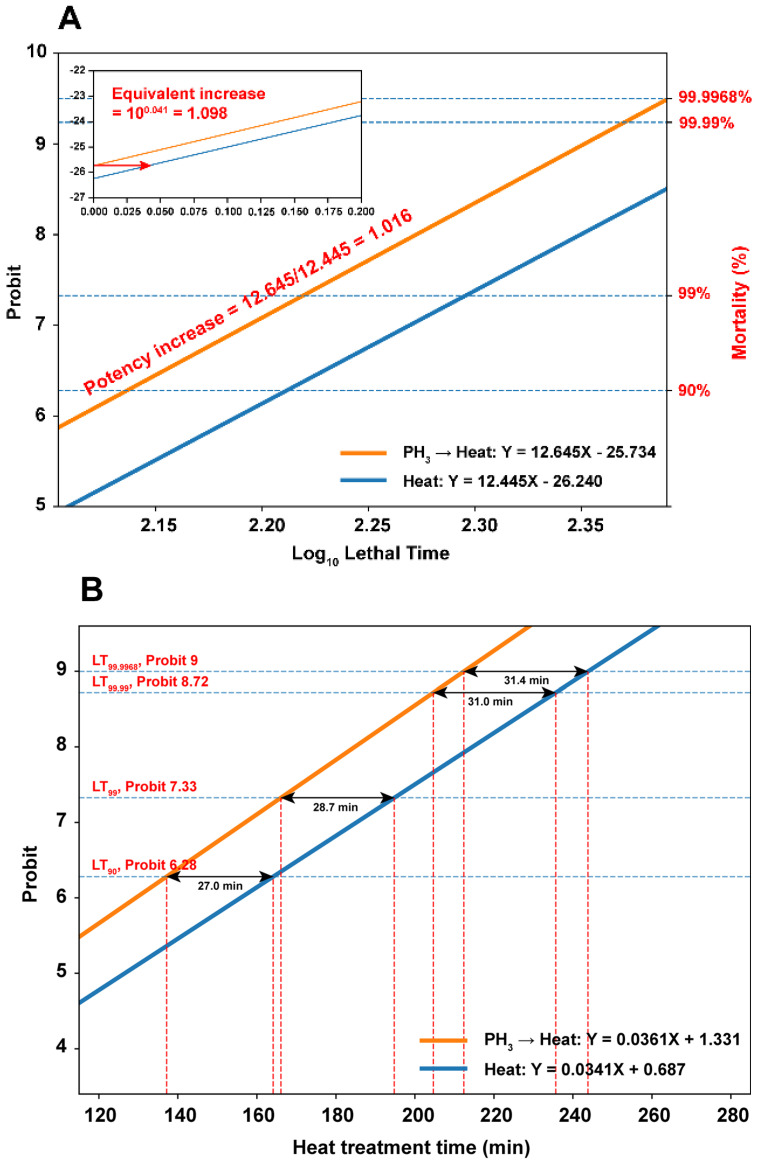
Probit-based comparison of heat treatment alone and phosphine followed by heat treatment against *Bactrocera correcta* eggs. (**A**) Estimation of equivalent time increase and potency increase based on probit regression models on the log_10_ lethal time scale. (**B**) Estimated time–mortality response curves showing LT_90_, LT_99_, LT_99.99_, and LT_99.9968_ gaps between the two treatments.

Comparative probit regression analysis further supported this enhancement effect for the selected 0.7 g m^−3^ PH_3_→Heat schedule (Figure 5). In Figure 5A, the PH_3_→Heat model showed only a slight increase in slope compared with heat treatment alone, with a slope ratio of 1.016. The equivalent horizontal shift between the two fitted response models corresponded to an equivalent increase of 1.098, indicating that heat treatment alone required approximately 1.20-fold longer exposure than PH_3_→Heat to achieve an equivalent mortality response. This suggests that PH_3_ pre-fumigation mainly enhanced heat-treatment efficacy by shifting the time–mortality response toward shorter exposure times, rather than by markedly changing the slope of the response [21,28].

The time-scale comparison in Figure 5B further illustrates this leftward shift by showing that the selected PH_3_→Heat treatment consistently reduced the estimated exposure time required to reach the selected mortality endpoints. The estimated time gaps between heat treatment alone and 0.7 g m^−3^ PH_3_→Heat were 27.0, 28.7, 31.0, and 31.4 min at LT_90_, LT_99_, LT_99.99_, and LT_99.9968_, respectively. The reduction at the Probit-9 endpoint was particularly relevant for quarantine treatment development, as it showed that PH_3_ pre-fumigation could shorten the heat exposure required to achieve high-level mortality [10,21]. Overall, these results demonstrate that PH_3_ pre-fumigation improved the efficiency of forced hot-air treatment by reducing the required treatment duration across both moderate- and high-mortality endpoints. Among the tested PH_3_ concentrations, 0.7 g m^−3^ PH_3_→Heat provided the most practical balance between reduced heat exposure, statistical separation at the high-mortality endpoints, and low fumigant input, and was therefore selected for large-scale confirmatory validation.

### 3.4. Large-Scale Confirmatory Trials

Large-scale confirmatory validation was performed to assess the efficacy of forced hot-air treatment alone and PH_3_→Heat against *B. correcta* eggs in mango fruit (Table 2). Heat treatment alone for 269.0 min resulted in zero survivors, with no live individuals recovered from 120 treated fruit containing an estimated 35,076 insects. PH_3_→Heat, using 0.7 g m^−3^ PH_3_ followed by 225 min of forced hot-air treatment, also resulted in zero survivors, with no live individuals detected from an estimated 36,936 treated insects.

The estimated efficacies were 99.9915% for heat treatment alone and 99.9919% for PH_3_→Heat. These values did not reach the Probit-9 level because the number of treated insects was lower than that required to statistically demonstrate 99.9968% mortality at the 95% confidence level. Specifically, 35,076 insects with zero survivors in the heat-only treatment and 36,936 insects with zero survivors in the PH_3_→Heat treatment provided approximately 67.1% and 69.0% confidence, respectively, for the exact Probit-9 threshold of 99.9968% mortality. At the standard 95% confidence level, these sample sizes support estimated mortalities of 99.9915% and 99.9919%, corresponding approximately to Probit 8.76 and Probit 8.77, respectively. Nevertheless, zero survival in both treatments provided strong operational support for the efficacy of the selected schedules against eggs, the most tolerant stage, in mango fruit under the tested validation scale. Although the present validation did not include a sufficiently large confirmatory population to provide formal statistical confirmation of Probit-9 efficacy at the 95% confidence level, PH_3_ pre-fumigation reduced the required heat-treatment duration by 44 min while maintaining zero survival, indicating that PH_3_→Heat remains a practically promising and shorter treatment schedule for phytosanitary disinfestation of mango fruit.

### 3.5. Fruit Quality Evaluation

**Figure 6:**
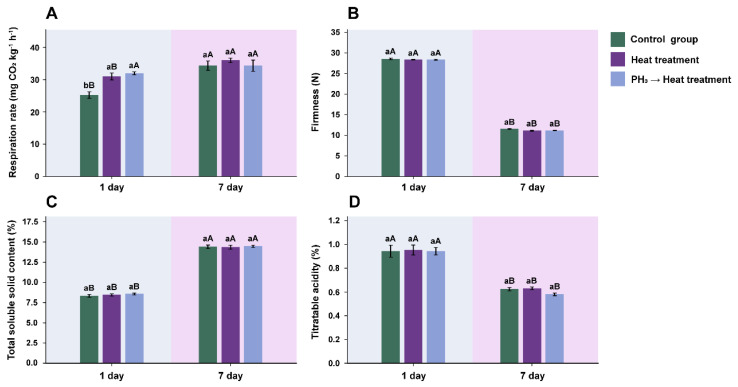
Changes in postharvest quality parameters, including respiration rate (**A**), firmness (**B**), total soluble solids (**C**), and titratable acidity (**D**), of mango fruit treated with forced hot air alone or phosphine followed by heat treatment. Different lowercase letters indicate significant differences among treatments within the same storage time, whereas different uppercase letters indicate significant differences between storage times within the same treatment (Tukey’s HSD test, *p* < 0.05).

The effects of forced hot-air treatment alone and phosphine followed by heat treatment (PH_3_→Heat) on mango fruit quality were evaluated during the post-treatment shelf-life period (Figure 6). Two-way ANOVA showed that respiration rate was significantly affected by treatment (*F* = 6.27, *df* = 2, 12, *p* = 0.0137), storage time (*F* = 33.88, *df* = 1, 12, *p* < 0.001), and their interaction (*F* = 4.31, *df* = 2, 12, *p* = 0.0388). At day 1, both heat treatment and PH_3_→Heat significantly increased respiration rate compared with the control, with mean values of 30.97 ± 1.08 and 31.93 ± 0.45 mg CO_2_ kg^−1^ h^−1^, respectively, compared with 25.20 ± 1.01 mg CO_2_ kg^−1^ h^−1^ in the control. However, by day 7, respiration rates had increased in all groups, and no significant differences were observed among the control, heat treatment, and PH_3_→Heat groups, indicating that the treatment-induced increase in respiration was transient.

Fruit firmness was strongly affected by storage time (*F* = 24485.67, *df* = 1, 12, *p* < 0.0001), but not by treatment (*F* = 2.72, *df* = 2, 12, *p* = 0.1059) or the treatment × storage time interaction (*F* = 0.26, *df* = 2, 12, *p* = 0.7760). Firmness decreased markedly from day 1 to day 7 in all groups, reflecting normal ripening-related softening during shelf life. At the same storage time, neither heat treatment nor PH_3_→Heat caused a significant reduction in firmness compared with the control.

TSS was also significantly affected by storage time (*F* = 1660.67, *df* = 1, 12, *p* < 0.0001), but no significant treatment effect (*F* = 0.36, *df* = 2, 12, *p* = 0.7022) or interaction effect (*F* = 0.14, *df* = 2, 12, *p* = 0.8669) was detected. TSS increased from 8.33% to 8.57% on day 1 to 14.37% to 14.47% on day 7, consistent with fruit ripening during shelf life. Similarly, TA was significantly influenced by storage time (*F* = 175.39, *df* = 1, 12, *p* < 0.0001), but not by treatment (*F* = 0.50, *df* = 2, 12, *p* = 0.6197) or the interaction between treatment and storage time (*F* = 0.30, *df* = 2, 12, *p* = 0.7449). TA declined from 0.94% to 0.95% on day 1 to 0.58% to 0.63% on day 7, with no significant treatment-related differences at either time point.

Overall, these results indicate that forced hot-air treatment alone and PH_3_→Heat did not adversely affect the main postharvest quality attributes of mango fruit during the shelf-life period. Although both heat-based treatments induced a short-term increase in respiration at day 1, this effect was no longer evident by day 7. Importantly, PH_3_→Heat shortened the required heat exposure for disinfestation without compromising firmness, TSS, or TA compared with heat treatment alone, supporting its potential as a shorter and fruit-compatible phytosanitary treatment for mango fruit.

## 4. Discussion

The present study showed that PH_3_-assisted forced hot-air treatment is a promising phytosanitary strategy for the control of *B. correcta* in mango fruit. Eggs were consistently identified as the most heat-tolerant stage, whereas first-instar larvae were the most susceptible. This result is biologically plausible because insect responses to heat are strongly stage-dependent and are influenced by differences in morphology, respiratory activity, developmental physiology, and stress-protective capacity [29,30]. Earlier work on tephritids and other quarantine pests has likewise shown that the most tolerant stage must be identified before a treatment schedule can be developed, and that eggs or late immature stages often define the phytosanitary target [5,7,18]. More broadly, studies on insect heat physiology indicate that tolerance under ramped heating conditions is not determined solely by absolute temperature, but also by the insect’s capacity to maintain metabolism and cellular protection during progressive thermal stress [31,32].

A major finding of this study is that PH_3_ pre-fumigation improved the efficacy of forced hot-air treatment mainly by shifting the time–mortality response toward shorter exposure times. The selected 0.7 g m^−3^ PH_3_→Heat schedule reduced LT_99.9968_ from 269.0 min for heat treatment alone to 224.5 min, while heat treatment alone required approximately 1.20-fold longer exposure to achieve an equivalent mortality response. Similar enhancement has been reported for PH_3_ followed by forced hot-air treatment in dragon fruit, but the broader significance of the present result is that it fits the general concept of combination phytosanitary treatments, such as CATTS, in which an additional physiological stressor reduces the thermal burden needed for quarantine security [12,18]. This shift suggests that PH_3_ pre-fumigation may have reduced the physiological robustness of the target stage, making the eggs less able to withstand the imposed heat stress. The current data also suggest that the enhancement was not simply concentration-dependent, because although all PH_3_→Heat treatments reduced LT_90_ and LT_99_, only 0.7 g m^−3^ PH_3_ produced a clearly distinct advantage at LT_99.99_ and LT_99.9968_. This plateau-like pattern resembles that observed in other synergistic postharvest systems and indicates that once a threshold of sensitization is reached, further PH_3_ does not necessarily improve the quarantine-relevant endpoint [13,33,34].

The mechanism underlying this enhancement likely involves more than simple additive toxicity. Previous phosphine studies have shown that PH_3_ can disrupt respiration-related processes and act as a broader regulator of insect stress resistance [1,35]. PH_3_ pre-fumigation may first reduce insect vitality through knockdown-like inactivity, temporary immobilization, or physiological debilitation, thereby weakening environmental stress resistance before heat exposure. These effects are closely related to respiratory metabolism, oxidative balance, mitochondrial energy production, neurophysiological processes, cellular repair, and stress recovery under adverse conditions [36,37]. In the most closely related PH_3_ + heat studies on *B. dorsalis*, forced hot-air treatment strongly induced HSP genes, whereas phosphine pre-fumigation suppressed this induction, and transcriptomic analysis further implicated HSP regulation, MAPK/ERK signaling, and stress-response pathways in the synergistic effect [38,39,40]. These molecular findings support the view that PH_3_ can compromise the heat-response capacity of fruit flies, particularly by limiting HSP-mediated cellular protection during subsequent thermal stress. Although no molecular assays were conducted here, the present results are consistent with the idea that PH_3_ weakens the capacity of *B. correcta* eggs to tolerate the subsequent thermal challenge, probably by interfering with cellular protection, respiratory metabolism, or repair processes before heat exposure. This sequence may explain why PH_3_ pre-fumigation promoted mortality during the following heat treatment, even though heat remained the major lethal factor. This interpretation is also in line with previous reports that phosphine can act synergistically with other fumigants in quarantine systems, including methyl bromide-based combinations [21,31,32].

The large-scale confirmatory validation supports the practical relevance of this treatment system. Both heat treatment alone and the selected PH_3_→Heat schedule resulted in zero survivors in mango fruit, providing strong operational support for the control of eggs, the most tolerant stage, under the tested validation scale. More importantly, PH_3_ pre-fumigation reduced the required heat-treatment duration by 44 min while maintaining zero survival.

This is commercially meaningful because heat-based quarantine treatments operate close to the tolerance limits of many commodities, and previous work in citrus, dragon fruit, and other fruits has shown that effective disinfestation must be balanced against the risk of heat injury and shortened shelf life [1,21,41]. At the same time, the treated populations in the present study were below the level required to statistically demonstrate Probit-9 efficacy at the 95% confidence level. The current validation should therefore be interpreted as strong operational support for the treatment, rather than definitive statistical proof at that level [13,20]. Compared with existing quarantine technologies, the PH_3_→Heat system developed in this study provides a complementary strategy that balances efficacy, treatment intensity, and commodity compatibility. Methyl bromide and methyl bromide-based combinations remain effective against tephritid pests, including *B. correcta*, but their use is constrained by ozone-depletion concerns and potential commodity injury [1,13]. Irradiation is a non-thermal phytosanitary option, but its wider commercial application can be limited by regulatory requirements, treatment facilities, and market acceptance [6,42]. Low-temperature PH_3_ fumigation has shown efficacy against *B. correcta* and other fruit flies, but longer exposure periods may be required for reliable control [10,41]. Conventional heat or vapor heat treatments are residue-free and widely accepted, but the required heat exposure often approaches the tolerance limits of fresh fruit [7,8,9]. In this context, PH_3_ pre-fumigation followed by forced hot-air treatment can reduce the thermal burden while maintaining mango fruit quality, providing a practical balance between quarantine efficacy and postharvest commodity protection.

Postharvest quality results further strengthen the value of the combined treatment. Most quality changes were driven by storage time rather than treatment, with firmness decreasing and TSS increasing as part of normal mango ripening. The only clear treatment effect was a transient increase in respiration at day 1 in both heat-based treatments, which disappeared by day 7. Thus, PH_3_ pre-fumigation did not impose additional negative effects on the principal quality attributes relative to heat treatment alone. This outcome agrees with previous phosphine or heat-treatment studies in loquat, mandarin, dragon fruit, and other fruit systems, where postharvest quality was generally maintained when treatment conditions were properly optimized [1,9,13,41]. It also suggests that, although mango and dragon fruit differ in ripening physiology and may not respond identically in respiration, PH_3_-assisted heat treatment can still be fruit-compatible [9,20,42]. A limitation of the present study is that the confirmatory trials did not exceed 100,000 insects and no mechanistic assays or broader quality indicators, such as color, aroma, or residue dynamics, were included. These aspects warrant further study. Overall, the present results support PH_3_ pre-fumigation followed by forced hot-air treatment as a technically feasible and commercially relevant phytosanitary option for mango fruit.

## 5. Conclusions

This study demonstrated that PH_3_-assisted forced hot-air treatment has strong potential as a phytosanitary disinfestation strategy for *Bactrocera correcta* in mango fruit. Eggs were identified as the most heat-tolerant stage, confirming their suitability as the target stage for treatment development. PH_3_ pre-fumigation enhanced the efficacy of forced hot-air treatment by reducing the exposure time required to achieve high mortality, with 0.7 g m^−3^ PH_3_ providing the most consistent and practically useful improvement at the quarantine-relevant endpoints. Under large-scale confirmatory validation, both heat treatment alone and PH_3_→Heat resulted in zero survivors, while the combined treatment reduced the required heat-treatment duration by 44 min. In addition, PH_3_→Heat did not adversely affect the major postharvest quality attributes of mango fruit during shelf life, indicating good commodity compatibility. Although the validation scale did not provide direct statistical proof of Probit-9 efficacy, the present results show that PH_3_ pre-fumigation followed by forced hot-air treatment is a technically feasible and commercially promising option for mango phytosanitation, and merits further evaluation with larger confirmatory populations and mechanistic studies.

## Figures and Tables

**Table 1:** Probit analysis for *Bactrocera correcta* eggs treated with forced hot air alone or PH_3_ followed by forced hot air in mango fruit.

Treatment	No. of Insects	Slope ± SE	Chi-Square	Heterogeneity	Probit Analysis of Lethal CT Values (95% CL)				SRs
					LT_90_	LT_99_	LT_99.99_	LT_99.9968_	
Heat	2520	12.445 ± 0.728	9.193	0.919	162.762 a (159.986, 165.585)	197.473 a (190.872, 204.303)	255.516 a (240.179, 271.832)	269.030 a (251.432, 287.859)	–
0.7 g m^−3^ PH_3_ →Heat	2520	12.645 ± 1.524	5.475	0.547	136.923 b (133.520, 140.326)	165.616 b (158.520, 172.712)	213.422 b (193.361, 235.564)	224.526 b (199.739, 249.313)	1.198
1.4 g m^−3^ PH_3_ →Heat	2520	10.555 ± 1.508	6.128	0.613	130.784 b (125.643, 136.136)	164.264 b (156.508, 172.405)	222.580 ab (196.149, 252.573)	236.524 ab (205.012, 272.881)	1.137
2.1 g m^−3^ PH_3_ →Heat	2520	10.333 ± 1.628	7.766	0.777	127.820 b (121.792, 134.148)	161.330 b (153.541, 169.514)	220.038 ab (191.933, 252.259)	234.129 ab (200.447, 273.470)	1.149

Note: Different lowercase letters within a column indicate significant differences at the 95% confidence level; confidence limit: CL; synergistic ratios: SRs.

**Table 2:** Large-scale confirmatory validation of forced hot-air treatment and phosphine followed by heat treatment against *Bactrocera correcta* eggs in mango fruit.

Treatment	PH_3_ Concentration	Treatment Time	No. of Test Fruit	No. of Larvae	Estimate Total Number of Treated Insects	Estimate Efficacy
Heat	–	269.0 min	120	0	35,076	99.9915%
Control	–	–	10	2923	–	–
PH_3_→Heat	0.7 g m^−3^	225 min	120	0	36,936	99.9919%
Control	–	–	10	3078	–	–

Note: Estimated total number of treated insects was calculated based on the infestation rate in control fruit and the number of treated fruit.

## Data Availability

The original contributions presented in this study are included in the article. Further inquiries can be directed to the corresponding authors.
